# Community, intervention and provider support influences on implementation: reflections from a South African illustration of safety, peace and health promotion

**DOI:** 10.1186/1471-2458-14-S2-S7

**Published:** 2014-06-20

**Authors:** Ashley van Niekerk, Mohamed Seedat, Sherianne Kramer, Shahnaaz Suffla, Samed Bulbulia, Ghouwa Ismail

**Affiliations:** 1 Medical Research Council-University of South Africa Violence, Injury and Peace Research Unit, Tygerberg, South Africa; 2 Institute for Social and Health Sciences, University of South Africa, Lenasia, South Africa

## Abstract

**Background:**

The development, implementation and evaluation of community interventions are important for reducing child violence and injuries in low- to middle-income contexts, with successful implementation critical to effective intervention outcomes. The assessment of implementation processes is required to identify the factors that influence effective implementation. This article draws on a child safety, peace and health initiative to examine key factors that enabled or hindered its implementation, in a context characterised by limited resources.

**Methods:**

A case study approach was employed. The research team was made up of six researchers and intervention coordinators, who led the development and implementation of the Ukuphepha Child Study in South Africa, and who are also the authors of this article. The study used author observations, reflections and discussions of the factors perceived to influence the implementation of the intervention. The authors engaged in an in-depth and iterative dialogic process aimed at abstracting the experiences of the intervention, with a recursive cycle of reflection and dialogue. Data were analysed utilising inductive content analysis, and categorised using classification frameworks for understanding implementation.

**Results:**

The study highlights key factors that enabled or hindered implementation. These included the community context and concomitant community engagement processes; intervention compatibility and adaptability issues; community service provider perceptions of intervention relevance and expectations; and the intervention support system, characterised by training and mentorship support.

**Conclusions:**

This evaluation illustrated the complexity of intervention implementation. The study approach sought to support intervention fidelity by fostering and maintaining community endorsement and support, a prerequisite for the unfolding implementation of the intervention.

## Background

The initiation, development, implementation and evaluation of community interventions are important for reducing violence and injuries in low- to middle-income contexts (LMICs), which are characterised by resource and capacity limitations. Although given interventions may appear effective by virtue of their content, a range of factors may affect the implementation of community-based interventions and impact upon intervention outcomes [1]. Successful implementation is therefore critical to attaining overall intervention outcomes [2-4]. The assessment of implementation processes thus gains significance in efforts to identify and better understand the factors that influence the realisation of intervention outcomes. There is a paucity of contextually relevant studies on influences specific to the implementation of injury prevention interventions in South Africa and other LMICs [5]. This article draws on a child safety, peace and health initiative to examine key local factors that enabled or hindered intervention implementation, in a context characterised by limited resources and uneven training and delivery capacities.

## Understanding implementation

The movement towards the utilisation of evidence-based practice continues to grow in several disciplines, including medicine and public health [e.g. [3,4,6]], the social sciences [e.g. [2]], and injury prevention [e.g. [7]]. The effective implementation of evidence-based interventions is complex, and is considered dependent on the dynamic interactions between a range of influences, including those from the social and political context, the organisational approach and capacities, the required and available resources, intervention recipient responses, service provider attributes and skills, stakeholder interactions, and implementation guidelines [e.g. [2,8-10]]. Accordingly a range of frameworks, taxonomies and classification systems have been developed to guide the effective implementation of interventions, and focused attention on key determinants or enablers of effective implementation. These frameworks include the Promoting Action on Research Implementation in the Health Sciences (PARIHS) [10], the Interactive Systems Framework for Dissemination and Implementation (ISF) [6], and the ecological framework developed by Durlak and DuPre [2].

Such frameworks collectively represent a critical evidence base for successful community intervention implementation. A review and meta-analysis of intervention implementation studies indicated that the implementation process is affected by variables related to communities, interventions and service providers, and aspects of the intervention delivery (i.e., organizational functioning) and support systems (i.e., training and technical assistance [2]. It is essential for the development and implementation of interventions in South African contexts, typified by fragmented community structures, heterogeneous identities and limited social and material resources [11], to consider the influences that may impact upon and promote or limit successful intervention implementation.

The community factors noted in the PARIHS [10] and ISF [6] models, and highlighted in the Durlack and Dupre review [2] include the influence of politics, funding, and policies that may inform the research and the existing theoretical paradigms that frame the research. The intervention itself needs to be sufficiently adaptable to meet the community priorities, and demonstrate contextual congruence. The intervention delivery system refers to general organisational factors, such as a shared vision and the ability to integrate the intervention into existing practices; specific processes, such as task formulation, decision-making and networking; and staffing considerations, such as leadership, intervention champions and administrative support. Provider characteristics include both the service provider’s perceived needs for, and the potential benefits of the intervention, as well as providers’ self-efficacy and skill proficiency. The intervention support system focuses on the training of service providers and the provision of resources such as skills, and emotional support to providers. Depending on the context and objectives of the intervention, different constellations of factors have been found to influence the implementation process [2,6,10].

These findings are supported by convergent evidence accumulating across various research domains, which confirms that implementation is a dynamic, albeit complex, developmental process that is subject to the influences of manifold interrelating factors at the levels of the individual, organisation and community [2,9,10]. These models serve as a platform for our analysis, which draws upon the experiences of the implementation of the South Africa-led Ukuphepha Child Study (UCS).

## Method

A case study approach was employed in an analysis of factors that governed the implementation of a community intervention, a component of a multi-intervention, multi-level child safety, peace and health promotion initiative, the UCS. Following the logic of the case study approach we used multiple data sources, namely author observations, reflections and discussions of the factors perceived to have influenced the implementation of the intervention [12,13].

### The intervention

The UCS is aimed at reducing injury-related risks and encouraging safety and health promoting behaviours and decisions amongst children and families living in low resource communities. The UCS is one of a number of interventions hosted by the Ukuphepha (Ukuphepha is an isiZulu word meaning demonstrating African safety), an initiative by partners from mainly African countries, that seeks the following: to implement, evaluate and maintain integrated safety, peace and health promotion programmes in resourced-challenged communities; to regularly convene scholarly fora dedicated to the generation of critical African-centred safety and peace promotion theories and methodologies; and to stimulate a network of service-based agencies across the continent [14].

The Ukuphepha initiative is currently developing sites for the study of innovative interventions that combine injury data collection with intervention applications and research-related community engagement, with research partners and community sites in South Africa, Mozambique, Egypt, Uganda and Zambia. The Ukuphepha focuses on safety, peace and health promotion interventions that are responsive to the injury profiles specific to participant communities [14]. The Ukuphepha serves as a basis for the longitudinal study of child, youth and elderly safety interventions, thereby strengthening the scientific basis of injury prevention and safety promotion initiatives in low-income, under-served communities [15]. The Ukuphepha involves various combinations of behavioural and environmental interventions that promote safety behaviours and incorporate the participation of stakeholders including community members, government, policy-makers and non-governmental organisations. These interventions are organised into three main intervention baskets: the Ukuphepha Child Study (UCS), which also includes a youth-centered multi-country Photovoice study [16]; the Spiritual Capacities and Religious Assets for Transforming Community Health by Mobilising Males for Peace and Safety (SCRATCHMAPS) study [17]; and a component on elder well-being and safety [16].

The UCS, which is being developed and piloted in South Africa over 2011-2014 is comprised of a suite of evidence-based interventions that promote child health and safety. The interventions have been implemented at three ecological levels, the home, early childhood development centres and the community [18]. The interventions use different combinations of educational activities, outreach programmes, advocacy activities, community mobilisation and the enhancement of community resources, and the development of first responders [14]. The community level interventions involved weekly campaigns that sequentially focused on the promotion of child development, promotion of family well-being and the prevention of child maltreatment, good child health including good nutrition and immunisation practices, and the reduction of priority injuries, including safe pedestrian behaviour, fire and burn prevention, and poisoning. The interventions were directed at caregivers and children.

The implementation in South Africa of the UCS’s community intervention provides the basis of the current case study analysis. This intervention was implemented between November 2011 and February 2012 in a low-income community in the Helderberg, on the periphery of Cape Town. The community consists of approximately 8000 residents of which 2700 are children. This community has limited infrastructure; 16% of the residents live in informal dwellings and close to 30% of the adult population is unemployed. The community suffers from a high incidence of injuries.

### Community engagement strategy

**Figure 1:**
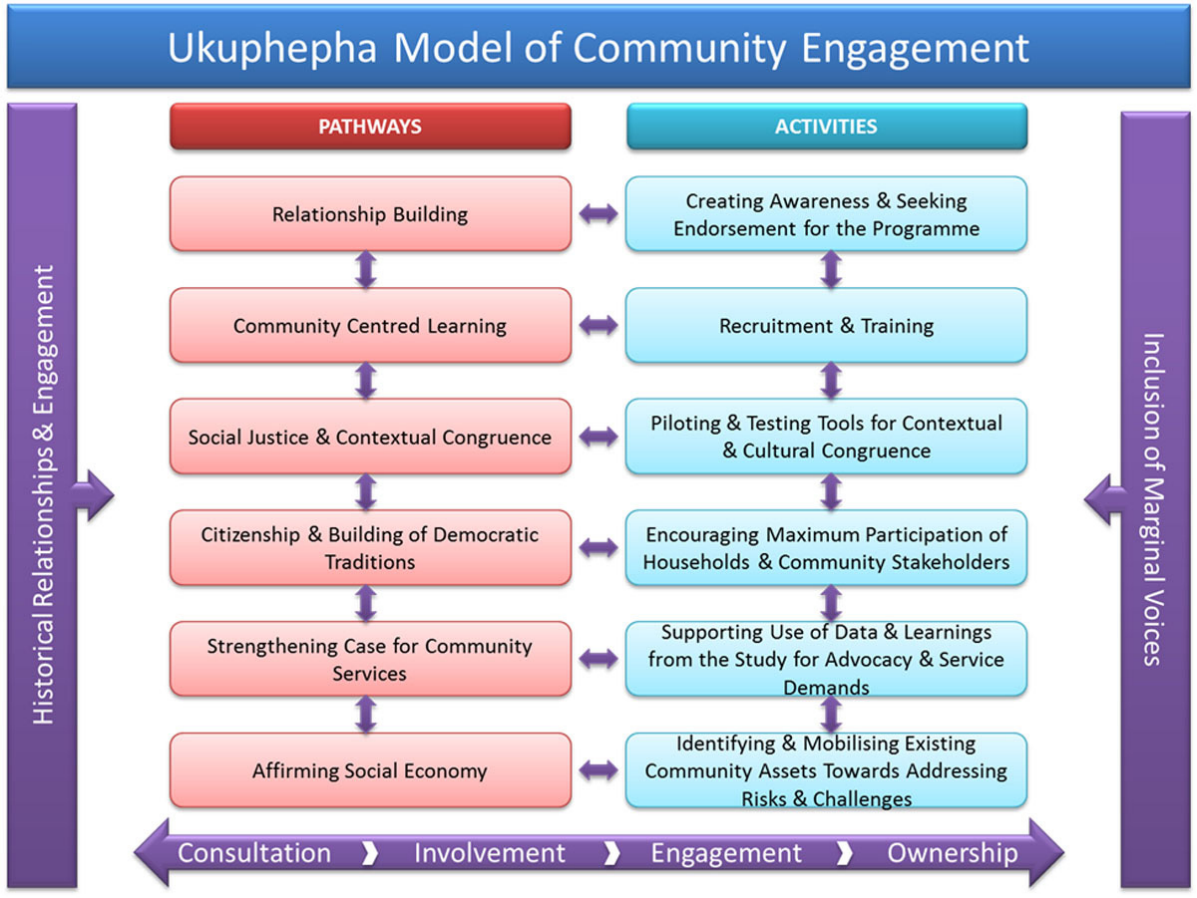
The Ukuphepha Community Engagement Model Source 18

The UCS utilised a community engagement strategy in order to ensure maximum community involvement in the implementation of the intervention (see Figure 1[18]). The UCS considers community engagement an integral element of effective community intervention implementation [18,19]. It frames community engagement as a dynamic and participatory process, and considers effective intervention implementation as contingent on community ownership. The engagement strategy drew on a number of pathways to foster community ownership, including the building of genuine relationships, the inclusion of marginal community voices, ensuring contextually congruent community-centred learning, promoting social justice and citizenship, and creating and aligning activities within democratic traditions. The UCS model operationalises the implementation process through a range of activities connected to each of the pathways referred to above. The pathways are not sequential but interactive. The UCS community engagement model indicates that relationship-building may be fostered through creating awareness and seeking active support for the intervention, and community-centred learning is encouraged through the recruitment of local residents and enhancement of community capacities to identify injury and health risks and recognise and promote local social economies. The social economy is further affirmed by enabling community residents to identify and mobilise existing community assets towards mitigating injury risks and strengthening protective factors. The UCS recruited and trained community members as local resources for data collection and intervention implementation. It encouraged social justice and contextual congruence through a process of piloting the content of the interventions for contextual and cultural relevance. Democratic traditions were supported by enabling maximum participation of residents and stakeholders at individual, household, and organisational levels. The case for community services and related advocacy activities were strengthened by promoting the use of supportive empirical data [18].

The implementation of the engagement strategy included multiple discussions with community leaders, community members and service stakeholders [18]. The UCS engaged with firstly community representatives and thereafter prospective service providers to determine the proposed intervention’s relevance to local needs and its potential benefits to the community, as well the envisaged participation and skills repertoire required for the effective delivery of the implementation. These discussions were followed by the recruitment and training of service providers, thereafter an intensive period for the development and finalisation of the intervention materials, followed by the implementation of the intervention itself. Parents, grandparents and children attended the interventions, conducted as a series of campaigns, with each having its own tailored programme. The intervention was complemented by the community-wide distribution of posters, flyers and newsletters on the child safety concerns highlighted in the community and possible and appropriate safety promotion responses.

### Research participants

The research team was made up of the six researchers and intervention coordinators, who led the development and implementation of the UCS in South Africa, and who are also the authors of this article. This team had been responsible for the conceptualisation, development implementation and analysis of the community intervention and its delivery, and comprised a multi-disciplinary group with extensive community psychology and public health qualifications and experience, with four having worked in the specific intervention community for more than a decade. This team therefore served as the primary informants to this case study, with their observations, reflections and notes on the implementation of the UCS providing the study data.

### Reflective observations and briefings

The research team drew from their field observations, notes and debriefing discussions with each other and the service providers. During the three month intervention delivery period, the UCS researchers recorded notes of their experiences, observations, reflections and discussions of factors perceived to have influenced the implementation of the intervention. This is considered to have enhanced and highlighted the transparency of the process and data dependability [20]. On conclusion of the intervention, the research team engaged in an in-depth and iterative dialogic process aimed at abstracting their experiences of the UCS [13]. This process involved a recursive cycle, initially from individual reflections, to group reflections and dialogue between the members of the research team. The team simultaneously drew from their field notes and provider discussions, thereby enabling further abstraction, the development of a consensus around leading issues, as well as the systematisation of the analysis in line with the PARIHS [10], the ISF [6] and Durlak and DuPre [2] models. According to Lincoln and Guba [21], extended engagement and continued observations favour the integrity and reliability of the research. This convergence in data sources is argued to strengthen research findings [13], and considered here to have promoted a deeper and fuller understanding of the implementation of the UCS.

### Analysis

The author reflections were informed by the field notes and weekly debriefing discussions, with the authors independently reflecting on their observations, notes and relevant documentation to identify key themes relevant to the analysis. The research team’s interpretations of the data were informed by their in-depth knowledge of the intervention, the implementation process, community setting and agents, and the body of knowledge on community-based interventions. Subsequent to this process, team meetings were convened during which the key emergent themes were reviewed, with points of convergence accepted, and divergence discussed further and reconciled. These discussions were guided by the existing intervention implementation analytic frameworks [2,9,10], with emerging themes reflecting key aspects of the implementation process.

## Influences on the implementation of the UCS

In the sections that follow, we reflect on the factors that were considered to have had an impact upon the implementation of the UCS. The sections focus on factors related to the community setting, the notable characteristics of the intervention, the intervention support system (i.e., training and technical assistance), and attributes of the community service providers.

### Community priorities and assumptions of identity

The important community factors identified in the literature include the contribution of politics, funding, and policy [2,10]. The UCS observations highlighted the following specific influences at the community level: the under-resourced nature of the community setting, the related competing social and occupational demands faced by the community, and the influence of UCS assumptions of the community’s identity.

#### Marginalisation and competing community priorities

The UCS was implemented in a South African community that is under-resourced and faced with a range of considerable obstacles when trying to access public health and social services [22,23]. Throughout the implementation of the UCS the authors observed that the community faced multiple demands and priorities related to employment, basic municipal services, welfare, health and family safety. In such settings, with extensive daily living pressures, including child care, family and household duties, families are also under extreme pressure to secure a sufficient income. These families and breadwinners often have precarious employment with little control over work schedules, which may include weekends. Other breadwinners may have more than one job, leaving minimal time for involvement in community activities [22,24]. Over the course of the UCS, the authors also noted sporadic episodes of tension and violence in the community, including gang-related violence, and the violence associated with the subsequent funerals of gang members, which disrupted several of the intervention events. During such periods, there was a palpable and understandable degree of fear amongst residents that prevented their active participation in the UCS.

In the UCS, community participation was therefore uneven, despite the multiple engagement pathways and extensive activities for promoting this engagement [18]. Adult participation in the intervention campaigns was minimal, and in some cases absent. Instead, the strongest and most consistent participation in the intervention activities was by unaccompanied children, who were observed to be keenly engaged in the campaign themes, through the interaction modalities used at the events [22]. The periodic threats of violence, in combination with the multiple pressures arising from the everyday demands of child care, family and household duties, and work responsibilities, restricted the extent to which residents could operate efficaciously as a community in intervention activities that required their active engagement [22].

#### Assumptions of community

The UCS had been framed within a conception of community that while defined as a geographic space within which residents share values, norms and activities that create a sense of community [e.g., [25]], are also characterised by both risk and protective characteristics, where protective factors may include a sense of community, community cohesion, community resilience and other similar capacities. These may be mobilised to mediate the deleterious consequences of risks and promote safety, peace and health [26]. The fostering of strong, protected, socially cohesive communities, which prioritise social connections and community life, is an important strategy for increasing safety [e.g. [27]].

The UCS conceptualises injury and violence prevention as a multi-disciplinary issue which is integral to the promotion of community safety, peace and health. Its integrated conceptual framework highlights: 1) notions of safety, peace and health as essential community resources; 2) the structural obstacles that function to obstruct individual and collective welfare; 3) change at the levels of beliefs, attitudes, values, knowledge, behaviours and structures within multiple levels; and 4) the enabling of individuals, collectives and broader systems to increase control over the determinants of injury and violence [14]. The UCS is accordingly based on a number of premises. Firstly, that child injury and violence prevention, an identified priority in the partner community, will gain maximum currency and impact if it is pursued within a comprehensive, child-centred safety, peace and health promotion initiative. Secondly, reductions in injury rates and severity, and the promotion of safety, peace and health are best achieved through addressing multiple-level drivers of injuries. Thirdly, a competency based approach that focuses on community assets, will reduce the risks for injuries and maximise promotive outcomes. Finally, community citizenship expressed through active forms of engagement is central to the realisation of these outcomes [14].

However, despite this conceptual, and the contextual and engagement assumptions of the UCS, we recognised underlying definitional difficulties associated with the term ‘community’. Whilst there is general academic consensus that ‘community’ denotes some form of group commonality, be it geographic, traditional, ethnic or vocational, the shared values of the group can never be static and unchanging. Communities are always in flux and understood as physical, social or intellectual milieus that accommodate multiple and often conflicting perceptions of these shared commonalities [1]. The presence or absence of cohesion, sense of belonging, efficacy and community norms are likely to shape the identity and sense of coherence that a community may experience [18].

The UCS’s conception of community also presupposed universal participation and thus adopted an engagement approach that strived to move from consultation to ownership for the purposes of obtaining optimal public participation in the community activities [18]. The limited funding and scope of the intervention however constrained the extent to which the project could be responsive to evolving community needs. Despite efforts to network and link the community to relevant resources, there were limited social and welfare services available for this, with consequent disappointments. The authors had also, based on a significant history of intervention work in the community, erroneously assumed that they had sufficiently understood the community dynamics and mood to obtain maximum participation. Whatever the community’s self-identity may be, the UCS had assumed that there was sufficient coherence, organisation, supportive social arrangements and sense of efficacy for the community to mitigate against threats to the implementation process.

### Intervention characteristics

Two intervention characteristics have been associated with effective implementation, namely intervention flexibility and compatibility (i.e. contextual appropriateness and congruence) [2]. The UCS observations highlighted the contributions of an inclusive approach, despite tensions between intervention implementation protocols and community needs.

#### Participatory structures and practices

The UCS was organised around a close working team comprising the research and intervention coordinators and the community service providers. The authors served as resource persons and trainers; they also led the implementation, and monitored and evaluated the intervention [see [28]]. While the overall scientific leadership was assumed by the authors, both authors and service providers developed protocols for implementation responsibilities, communication processes, coordination with broader community systems, and administrative support [see [29]]. The overall protocols were implemented by the coordinator of the provider team, making available daily, weekly and monthly operational and attendance reports.

The UCS involved weekly campaigns with each intervention event jointly formulated and designed by the community service providers and the authors. This team managed the campaign programme, messaging, and implementation strategy. The UCS also involved other agencies to enhance cooperation and collaboration between relevant stakeholders, and which in turn also contributed different perspectives, skills, and resources to the UCS implementation.

Regular consultation and debriefing on the implementation of the UCS afforded the community service providers with opportunities for reflection on their experiences and challenges. This inclusivity or participatory process engendered and supported an ethos or sense of citizenship, and the building within the UCS team of democratic practices. The reflexivity that this encouraged illuminated and strengthened the compatibility between the intervention and community priorities and values. Shared decision-making around planning, practical implementation, and organisational management was intensive and broad, with the authors, in the immediate run-up to the campaigns, present in the community daily, to monitor implementation and address emerging queries and challenges. This informed the authors about intervention and implementation concerns, and stimulated discussions about the possible adaptations required to enhance the contextual appropriateness of the UCS.

Overall, these discussions indicated that the intervention was regarded as congruent with the community’s child safety agenda. There were, however, some variation in provider reports of the compatibility and adaptability of the UCS’s thematic focus. The providers indicated that, for example, the lack of a targeted focus on alcohol and drug use, given its prevalence in the community and its impact on local child safety, reduced the extent of the intervention’s synergy with the priorities of the community [22]. The service providers also contested the inclusion and significance of poisoning prevention, indicating this as a lesser priority despite other previous indications to the contrary [23].

#### Tensions between implementation protocols and community needs

The adherence by the UCS to an evidence-based implementation protocol limited the space for community and intervention recipient inputs. Community stakeholders sought a larger participatory space through which they could inform the implementation protocol and process. The service providers suggested that the UCS, regardless of its historical and cultural immersion, may not have understood the nuanced features and needs of the community. This reflects the tension between observance of evidence-based implementation protocols and community needs, even within the context of a developed participatory engagement process [30]. Jensen and colleagues [30] argue for a shift in emphasis from what works under optimal research conditions to “what works that is also palatable, feasible, durable, affordable, and sustainable in real world settings” (p. 206). This disjuncture seemed to have exacerbated the power dynamic between the UCS and the community. Despite the authors’ awareness of the potential for the community to experience the research and intervention coordinating team as the experts by virtue of their access to theory, resources and knowledge-legitimating mechanisms [17], the attempt to adhere to the UCS implementation protocol contributed to an insider-outsider experience. This dyad was accentuated by the context, which is characterised by socio-economic disparities, racialised power structures, and cultural, linguistic and ethnic differences [31]. Despite the UCS commitment to recognise local knowledge, it appeared that community members were not accorded sufficient status in the UCS as local knowledge brokers and experts [18].

The tension between intervention implementation fidelity and adaptability echoed the broader tensions inherent in applying a structured intervention protocol in a community setting [30]. Community-based research however does value context-driven, participatory and asset-based research [see [32]] and indigenous or local knowledge in the knowledge construction agenda [33]. The recent call to merge empirical, evidence-based and community-based practices [6,34] underscores the need to bridge the gap between these traditions in order to ensure the identification of effective interventions that can demonstrate a high level of quality [35]. Durlak and DuPre [2] have indicated that organizations have a better chance of effective implementation of interventions with some degree of flexibility than those that must be conducted strictly by protocol. The UCS experiences indicate that its adherence to an evidence-based intervention protocol is possible within a supportive community engaged approach.

### Service provider characteristics and expectations

The UCS employed a service provider team that comprised of residents from the local community and appointed a coordinator from this team to oversee the overall data collection and intervention process. In turn, the intervention provider team selected two co-coordinators who, on a rotational basis, supported the facilitation of intervention events. The authors offered oversight and reviewed the implementation plans and activities to ensure that they were consistent with the intervention objectives and the supporting evidence base. The planning of all events was jointly formulated and designed by the intervention providers and the authors. This team together managed the content design, messaging, implementation strategy, and oversaw the coordination of the intervention activities. This coordinating strategy promoted a sense of ownership.

The intervention delivery system was structured as a learning entity [36], so on a weekly basis a review process was conducted involving the providers and authors, which promoted reflections and learnings for continuous improvement. The providers, based on their long-term residency, were sufficiently attuned to local norms. The uneven levels of community engagement described earlier seem to be partially related to the capacities of the intervention delivery system. The local intervention coordinators were at times unable to demonstrate the requisite leadership and coordination skills required to mobilise and encourage broader community involvement and participation in the intervention. Providers were unable to implement strategies to have adult community members (especially) avail themselves for events and meetings on specified days to facilitate synergy between community priorities and intervention objectives. However, the providers were able to use their skills to mobilise children, the main recipients of the intervention [22,36].

In general, the providers expressed a sense of affirmation from the recognition of their life experiences and the opportunities for maximum participation during the training phase. The providers indicated that they appreciated the combination of interactive formats that encouraged exploration and participatory learning [see [36]], by deepening their understandings of injury prevention through personal experience, interpersonal connections, and the development of tolerance for differences of opinion. This participatory process is argued to engender an ethos of citizenship and the building of democratic traditions [18]. During the training, providers shared their own content-related stories about burns, violence and injuries, a process that they reported to be acknowledging of their life experiences and local knowledge. The providers reported that this process of sharing exemplified an effort to promote collaboration, community ownership and the prioritisation of local needs. Consistent with other studies [see [37,38]], the use of modelling, role play and performance feedback encouraged active participation. Recent studies suggest that collaborative endeavours may increase intervention effectiveness [see for example [39]].

### An intensive training and support system

The UCS offered a support system that focused on the training of service providers and the provision of resources such as skills, and emotional support [2]. The UCS offered all providers intensive training in the extent, occurrence, risk and prevention of priority childhood injuries (including those due to burns, traffic, poisoning, maltreatment), and the promotion of selective health practices [40]. The capacitation of local coordinators and intervention providers also focused on a range of skills (e.g. first aid, leadership and facilitation). The latter involved a series of training modules, which utilised role-play and other interactive methods, and was also accompanied by an assessment to ensure that the trainee interventionists were competent in the intervention content and the required implementation activities. The training was designed to prepare the providers for the coordination and delivery of the safety intervention, as well as for the mobilisation of community participation [36].

The providers reportedly experienced the training courses intended to prepare them for implementation as an expression of community centred co-learning. The sense of community centred learning arose from the providers’ perceived sense of relevance and beneficence of the course. They reported that the training had been aligned to the priorities of their community, but queried the prioritisation of the paraffin poisoning prevention focus, indicating that this was less of an issue for this community. The providers also identified other issues, such as alcohol and drug use, as relevant intervention foci that should have been accommodated in the intervention.

The providers reported that the training contributed to an increased sense of self-efficacy and confidence [see [28,41]]. However, funding constraints and stringent timeframes imposed by the project funder limited the length of the training offered to the providers. The research team members served as mentors to the providers, and ensured that technical and emotional support was provided on a weekly basis in the form of group debriefings. This was reported by the providers as essential for the purposes of effective intervention coordination and implementation [see [29]]. These debriefing sessions were supplemented with regular meetings between providers and community coordinators. The UCS support system encouraged cohesion and connectedness between providers and the research team, likely contributing to the minimal drop-out by providers. Nonetheless, the research team was occasionally called on to facilitate the resolution of conflict between providers, and administrative challenges such as the efficient processing of remuneration claims. The research team engaged in weekly discussions to review issues arising from training, intervention event execution and provider functioning.

The research agency provided the necessary resources for the implementation of the intervention. These included communication and audio-visual support, transport, meals, stationary, identifying clothing and cards, training materials and equipment, and other incidentals. All providers were remunerated with a nominal allowance. Additionally, the intervention mobilised local safety promotion stakeholders to demonstrate safety practices and behaviours, such as the construction of safe home and recreational spaces [see [29]].

The provision of training positioned the providers as resource persons supported by the UCS. This positioning produced a tension in the providers’ identity; they were viewed as community members and at the same time as providers who had access to particular resources. Those who were not selected for training as providers may have felt excluded from the benefit of training processes and resources. Such concerns may be a matter of unintended skills or resource stratification, and may potentially undermine the implementation of community-based interventions [2,42].

## Conclusions

The intervention sought to enhance child safety, peace and health through the mobilisation of existing community assets and resources, and the implementation of a community-based intervention that draws on the principles of evidence-based intervention and community-based participatory research [14,18]. Our analysis of the UCS highlighted key local factors that influenced its implementation. These included the degree of intervention responsiveness to the local context; a coherent and comprehensive community engagement strategy; intervention synchronisation with provider perceptions of its pertinence and value; high intervention compatibility with the local community and adaptability potential; shared decision-making in intervention delivery processes; and intensive community-centred training and learning, and ongoing mentorship.

However, our evaluation demonstrated challenges to the implementation fidelity of the UCS, as manifest in varying prioritisation of intervention themes and uneven recipient receptiveness. Durlak and DuPre [2] have argued that fidelity and adaptation should not be dichotomised or understood as exclusive to one another. Rather, the measurement of both aspects should result in an appropriate fusion whereby core intervention components are maintained and less central elements are adapted to ensure ecological appropriateness and improved local effectiveness. A number of studies have demonstrated improved intervention outcomes as a result of adaptation exercises [see [43-45]]. In view of the growing recognition that prevention interventions should be contextually congruent, it becomes necessary for interventions to accommodate community concerns and needs, especially as this is imperative for increased community ownership and accountability [46]. The UCS approach therefore sought to address intervention fidelity and adaptability by assuring the primary objectives of safety, health and peace promotion, whilst sustaining its commitment to foster and maintain community endorsement and support, a prerequisite for the ongoing implementation of the intervention.

## Competing interests

The authors declare that they have no competing interests.

## Authors' contributions

AVN contributed to the conceptualisation, drafting and editing of the entire manuscript. MS led on the conceptualisation of the intervention and manuscript, and contributed to its development and finalisation. SK developed the initial draft of the manuscript. SS contributed to the conceptualisation, drafting and editing of the entire manuscript. SB co-supervised the implementation of the intervention and contributed to the drafting of the analysis and discussion. GI assembled relevant information for purposes of this evaluation and drafted the Methods section. All authors were involved in the data collection process, and read and approved this version of the manuscript.

## Declarations

The implementation of the UCS and this case study was supported by the University of South Africa and the South African Medical Research Council.

This article has been published as part of *BMC Public Health* Volume 14 Supplement 2, 2014: Responsive health systems: working with the community on control of non-communicable diseases (NCDs). The full contents of the supplement are available online at http://www.biomedcentral.com/bmcpublichealth/supplements/14/S2.
